# Commuter exposure to fine and ultrafine particulate matter in Vienna

**DOI:** 10.1007/s00508-017-1274-z

**Published:** 2017-10-09

**Authors:** Georg Strasser, Stefan Hiebaum, Manfred Neuberger

**Affiliations:** Center for Public Health, Medical University of Vienna, Kinderspitalgasse 15, 1090 Vienna, Austria

**Keywords:** Air pollution, Fine particulate matter, Ultrafine particles, Lung deposited surface area, Commuting

## Abstract

Mass concentrations PM_10_, PM_2.5_, PM_1_, particle number concentrations of ultrafine particles and lung deposited surface area were measured during commutes with a subway, tram, bus, car and bicycle in Vienna for the first time. Obtained data were examined for significant differences in personal exposure when using various transport modalities along similar routes. Mean PM_2.5_ and PM_1_ mass concentrations were significantly higher in the subway when compared to buses. Mean PM_10_, PM_2.5_ and PM_1_ mass concentrations were significantly higher in the subway when compared to cars using low ventilation settings. Particle number concentrations of ultrafine particles were significantly higher in trams when compared to the subway and lung deposited surface area was significantly greater on bicycles when compared to the subway. After adjusting for different vehicle speeds, exposure to PM_10_, PM_2.5_ and PM_1_ along the same route length was significantly higher in the subway when compared to cars while exposure to ultrafine particles and partly also lung deposited surface area was significantly higher in bus, tram and on bicycle when compared to the subway. Car and bus passengers could be better isolated from ambient fine particulate matter than passengers in the subway, where a lot of ventilation occurs through open windows and larger doors. Tram passengers and cyclists might be exposed to increased amounts of ultrafine particles and larger lung deposited surface area due to a closer proximity to road traffic. Comparing cumulative exposure along the same route length leads to different results and favors faster traffic modes, such as the subway.

## Introduction

Evidence suggests that short and long-term exposure to fine particulate matter (FPM) is associated with increased long-term [1, 2] and short-term [3, 4] mortality. Associations with ultrafine particles (UFP) are weaker, possibly due to lack of comparable data and high variability of particle number concentration (PNC) in space and time [5] as well as the small number of studies available on lung deposited surface area (LDSA), a proxy for surfaces of UFP which come into contact with the cells in the respiratory tract.

In Vienna, increased levels of PM_10_ (particulate matter ≤10 µm) and PM_2.5_ have been shown to predict all-cause mortality [6] and PM_2.5_ has been linked to hospital admissions due to respiratory symptoms [7]. Motor traffic is one of the main contributors to atmospheric FPM in European cities [8]. Traffic-generated particles could have a stronger impact on mortality than particles from coal combustion or crustal particles [9]. Commuting via public or private transport tends to occur near urban traffic which leads to a considerably higher exposure to particulate air pollution. Numerous studies have tried to assess if and how choice of transport mode can impact personal exposure to FPM and UFP and have partly shown conflicting results. Exposure differences often vary when comparing mass/number concentrations of fine and ultrafine particles.

A recent study in Milan comparing walking, cycling, commuting by car and by subway found the highest mass concentration (MC) levels of fine particles in the subway followed by bike, walking and car whereas the PNC of UFP was highest while cycling, followed by walking, taking the subway and the car [10]. In Beijing PM_2.5_ levels were found to be highest in the subway, followed by walking, surface railway and bus, whereas PNC levels of ultrafine particles were highest in air-conditioned bus, followed by surface railway, subway, walking and non-air-conditioned bus [11]. Results obtained in a study comparing bus, bicycle, car and subway in Santiago de Chile showed highest MC levels of PM_2.5_ in bus followed by subway, bicycle and car and PNC levels of UFP highest in bus, followed by bicycle, car and subway [12].

A study done in Barcelona comparing bus, tram subway and walking found higher PM_2.5_ MC levels in subway and bus, compared to tram and highest PNC levels of ultrafine particles in the bus, followed by tram and subway [13]. In Hong Kong, PM_10_ exposure was shown to be highest in non-air-conditioned roadway transport, followed by marine transport, air-conditioned roadway transport and railway transport [14]. Results of a study done in Arnhem, The Netherlands, showed higher PM_10_ levels in bus, followed by car and bicycle and higher PM_2.5_ levels in cars than in buses and on bicycles [15]. Drawing conclusions for other cities out of the growing pool of personal exposure studies is difficult due to differences in local city characteristics, such as weather, local emissions and properties of public and personal transport vehicles.

We conducted the first field study in Vienna, the capital of Austria, which has 2.6 million inhabitants in its metropolitan area, to explore for differences in personal exposure to PM_10_, PM_2.5_, PM_1_ and UFP/LDSA in subway, bus, tram, car and on the bicycle.

## Material and methods

### Data collection

**Fig. 1:**
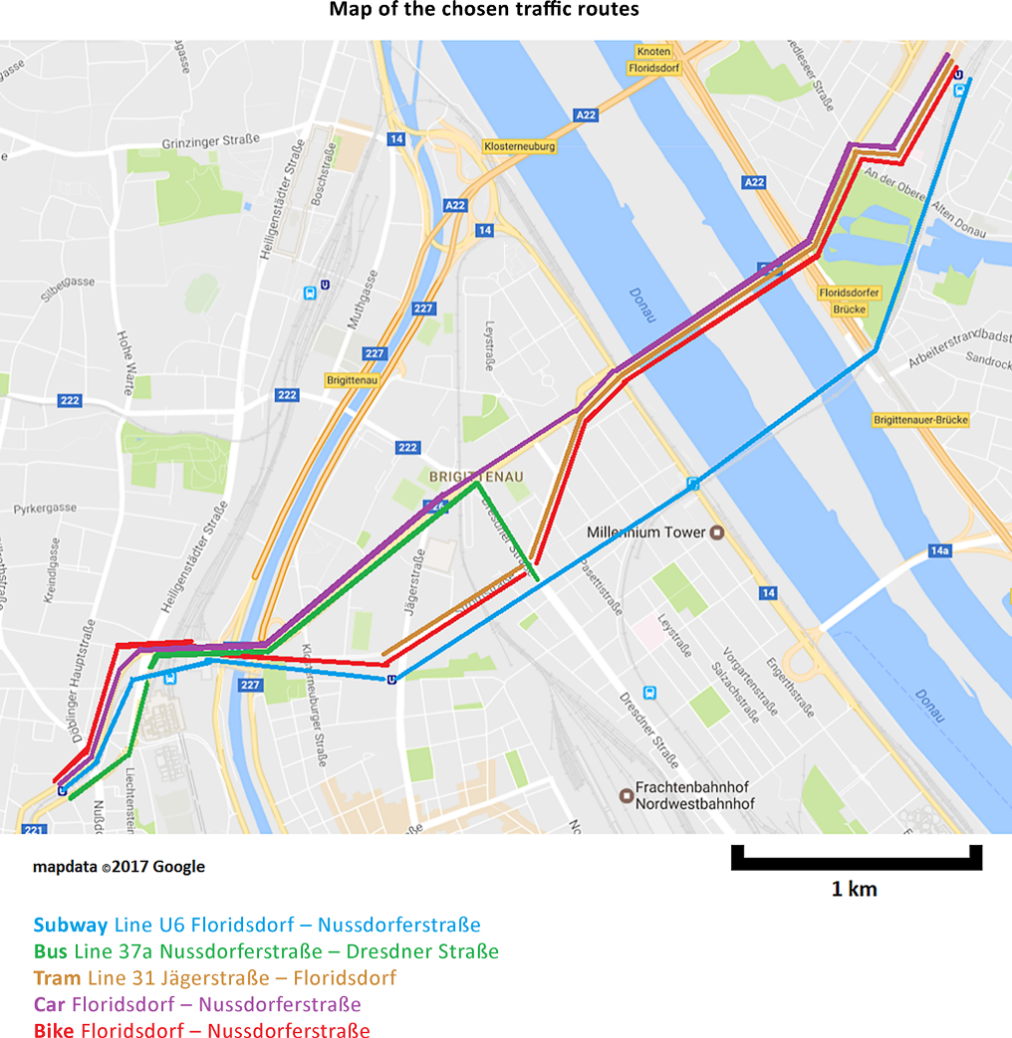
Map of the chosen traffic routes

We obtained mass concentrations of PM_10_, PM_2.5_, PM_1_ as well as particle number concentrations of ultrafine particles and LDSA using portable particle counters during commutes along typical commuter routes shown in Fig. 1 on 7 days between October 2015 and June 2016. The chosen area is typically utilized by commuters going to work in the inner city from the northeast urban and suburban parts of Vienna. The existence of busy streets and so many highly frequented public transport lines within a relatively close distance is unique in this part of Vienna and invites comparisons between traffic modes.

Measurements were done once or twice between 08:00 and 12:00 at time intervals of 2 h during which we consecutively used all transport modes once. Background levels of PM_10_ and PM_2.5_ during the respective time periods were obtained using local pollution data provided by the city government (Municipal Department 22). Mean ambient mass concentrations shown in Table 1 were calculated using data from three stationary measuring devices at the Viennese General Hospital (N48° 13′ 10.3″ E16° 20′ 44.0″), Stadlau (N48° 13′ 34.9″ E16° 27′ 30.0″) and Taborstraße (N48° 13′ 0.3″ E16° 22′ 51.3″).

**Table 1:** Background levels of PM10 (PM10-ba) and location of measurements of fine particulate matter (FPM) and ultrafine particulate matter (UFP)

M	Date	PM_10_-ba (µg/m^3^)	FPM data	UFP data
1	27.10.2015	29.1	s, b, t, c	s, b, t, c
2	17.11.2015	27.8	–	s, b, t, bi
3	23.02.2016	33.7	s, b, t, c	s, b, t, c
4	25.02.2016	19.9	s, b, t	s, b, t
5	25.02.2016	19.9	s, b, t	s, b, t
6	01.03.2016	14.0	s, b, t, c	s, b, t, c
7	01.03.2016	14.0	s, b, t, c	s, b, t, c
8	02.06.2016	10.8	s, b, t, bi	s, b, t, bi
9	02.06.2016	10.8	s, b, t, bi	s, b, t, bi
10	06.06.2016	19.5	s, b, t, bi	s, b, t, bi
11	06.06.2016	19.5	s, b, t, bi	s, b, t, bi

*M* Measurement, *s* subway, *b* bus, *t*: tram, *c* car, *bi* bicycle, *PM10-ba* background levels of PM_10_, *FPM* fine particulate matter, PM_10_, PM_2,5_ and PM_1_ were measured in/on the respective traffic vehicles, *UFP data* ultrafine particles and LDSA were measured in/on the respective traffic vehicles

### Characteristics of transport vehicles

The chosen section of the subway line runs underground for approximately two thirds of the route. Trains (Bombardier® T, T1) were partially air-conditioned and mostly ventilated via open windows. Buses (MAN Lion’s city®, Mercedes-Benz Citaro® 2) were air-conditioned and powered by diesel engines. Trams (types A, B, E1, E2) were not air-conditioned and ventilated via open windows. Test cars (Audi A3® 1997, Mazda 5® 2006 and Ford B‑Max® 2015), were all gasoline-powered and air-conditioned with air flow manually set to low. Windows were closed and the cabin was ventilated with ambient air. The bike route runs mostly directly next to traffic. Measuring devices were placed on passenger seats 2 m away from entry doors in public transport vehicles, on the left rear passenger seat in cars and strapped to the test person’s back with air inlets at breathing level on the bicycle.

### Measuring devices

We measured PM_10_, PM_2.5_ and PM_1_ mass concentrations of fine particles in 6‑s intervals using the portable optical particle counter Grimm Aerosol Spectrometer®, Model 1.108 (GRIMM Aerosol Technik Ainring GmbH & Co. KG, Ainring, Germany), the performance of which has been evaluated before [16] and which detects particles with an aerodynamic diameter over 300 nm. Number concentrations of ultrafine particles between 300 and 10 nm were obtained in 1‑s intervals using the miniDISC® diffusion size classifier (Dr. Martin Fierz, Fachhochschule Nordwestschweiz, Windisch, Switzerland) and LDSA was estimated according to the International Commission on Radiological Protection (ICRP) [17].

### Statistics

The FPM, UFP and LDSA data were summed up into one mean for each commute. We observed minimal differences in traffic and background pollution levels during the 2‑h measuring intervals and treated the consecutively obtained means as simultaneously measured data points. After testing all samples for Gaussian distribution with Kolmogorow-Smirnow tests we used Friedman’s analysis of variance by rank for dependent samples to test for significant differences between mean FPM and UFP mass/number concentrations and LDSA. When significant differences were indicated, post hoc analyses were done using Dunn-Bonferroni tests.

#### Adjusting for different velocities

We compared FPM, UFP and LDSA data with and without adjusting for different speeds, which was done by dividing the obtained data by the calculated mean speeds of the respective traffic modes, i. e. 7.6 m/s for subway, 4.6 m/s for bus, 5.8 m/s for tram, 6.5 m/s for car and 4.5 m/s for bicycle.

## Results

We obtained the set of data shown in Table 1. We were not able to do measurements in every single transport vehicle every time. Using the available data we did three seperate comparisons. Comparison one was done between subway, bus and tram. We used all 11 measurements to explore for differences in UFP exposure and all but measurement 2 to explore for differences in FPM exposure. In the second comparison the car was included. We used measurements 1, 3, 6 and 7 to explore for differences in UFP and FPM exposure. The third comparison was between subway, bus, tram and bicycle. For FPM comparisons we used measurements 8–11 and for UFP comparisons we additionally used measurement 2.

Table 2 shows obtained median mass concentrations of PM_10_, PM_2.5_ and PM_1_, as well as particle number concentrations of ultrafine particles and LDSA.

**Table 2:** Median mass concentrations (PM), particle number concentrations (PNC) and lung deposited surface area (LDSA)

	PM_10_ (μg/m^3^)	PM_2.5_ (μg/m^3^)	PM_1_ (μg/m^3^)	UFP ﻿(pt/cm^3^)	LDSA (μm^2^/cm^3^)
*Comparison 1*					
Subway	58.1	19.9 _*1*_	12.7 _*2*_	7233.3 _*3*_	3.56
Bus	35.2	8.2 _*1*_	5.8 _*2*_	12296.0	5.05
Tram	37.0	13.3	8.1	11783.2 _*3*_	5.01
*Comparison 2*					
Subway	72.7 _*4*_	21.9 _*5*_	11.4 _*6*_	6480.5	5.99
Bus	27.8	6.2	3.8	10598.3	6.46
Tram	51.1	14.8	6.9	10008.2	5.01
Car	19.2 _*4*_	4.5 _*5*_	3.0 _*6*_	8848.4	3.33
*Comparison 3*					
Subway	50.0	19.5	13.3	8608.9	4.00 _*7*_
Bus	41.4	9.1	6.0	13129.9	6.32
Tram	73.2	14.9	8.0	13311.5	7.49
Bicycle	16.7	9.5	7.4	18199.6	35.47 _*7*_

Significant differences in exposure between traffic modes are indicated by matching subscripts, e.g. exposure to PM 2,5 was significantly lower in the bus than in the subway in the first comparison, exposure to PM 1 was significantly lower in the car than in the subway in the second comparison

### Subway vs. bus vs. tram

**Fig. 2:**
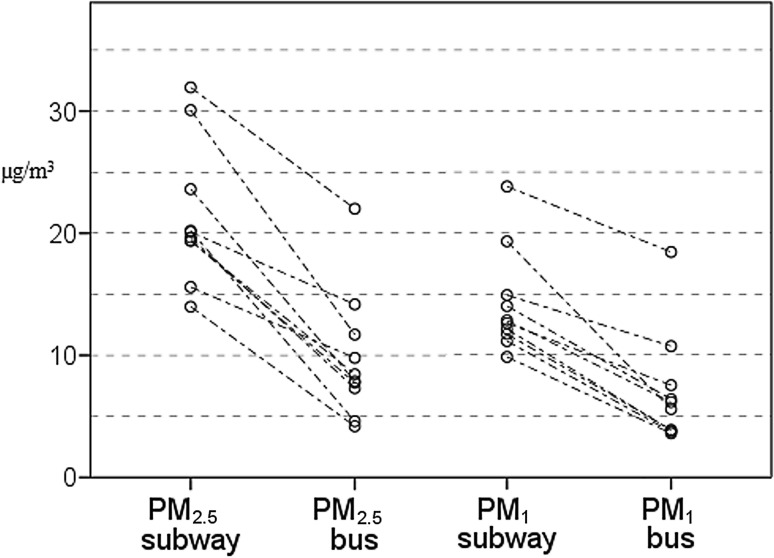
PM_2.5_ and PM_1_ data for subway and bus measurements

We found significantly higher median MC (µg/m^3^) of PM_2.5_ and PM_1_ in the subway compared to the bus (19.9 vs. 8.2, *p* < 0.001 and 12.7 vs. 5.8, *p* < 0.001, respectively). Fig. 2 shows that PM_2.5_ and PM_1_ mass concentrations were consistently lower in all 10 measurements.

**Fig. 3:**
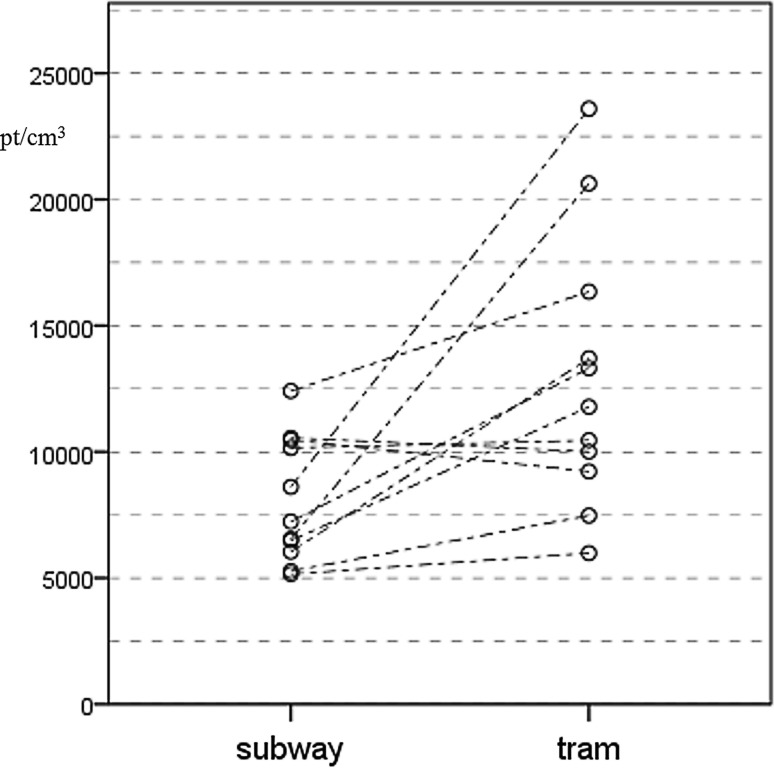
Ultrafine particle number concentration data of subway and tram measurements, UFP exposure was higher in the tram during most measurements

The mean PNC of ultrafine particles (pt/cm^3^) was higher in the tram when compared to the subway as (11,783 vs. 7233, *p* = 0.017). Individual measurements are shown in Fig. 3.

No statistically significant differences were observed for PM_10_ mass concentrations and LDSA.

### Subway vs. bus vs. tram vs. car

**Fig. 4:**
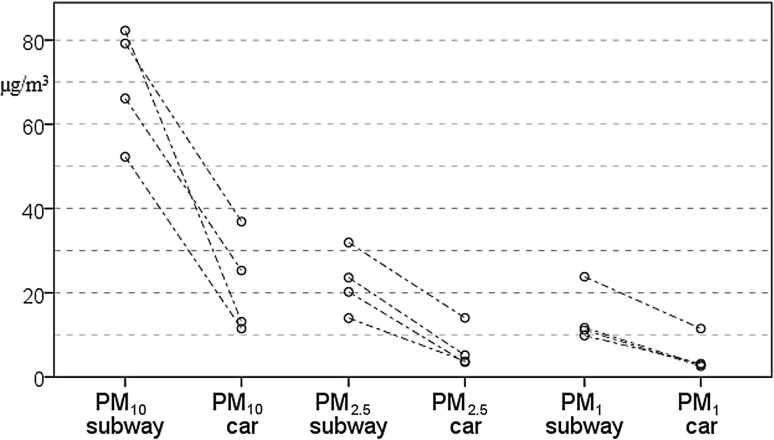
PM_10_, PM_2.5_ and PM_1_ data for subway and car

Mass concentrations of PM_10_, PM_2.5_ and PM_1_ (µg/m^3^) in the subway were more than three times as high as in the car (72.7 vs. 19.2, *p* = 0.016, 21.9 vs. 4.5, *p* = 0.006 and 11.4 vs. 3.0, *p* = 0.006, respectively). Fig. 4 shows that PM_10_, PM_2.5_ and PM_1_ levels were consistently lower in the car compared to the subway. No significant differences were observed for UFP and LDSA.

### Subway vs. bus vs. tram vs. bicycle

**Fig. 5:**
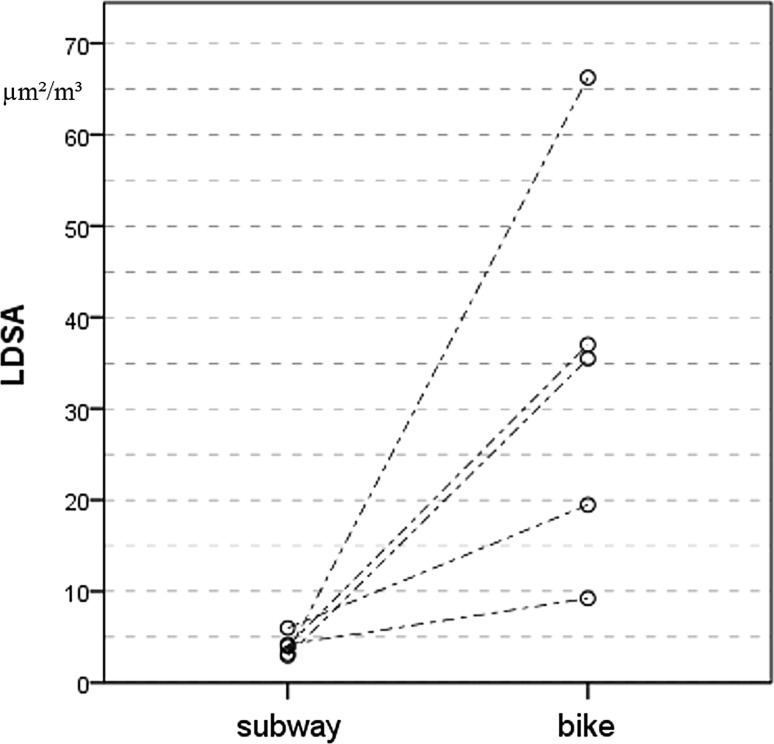
Lung deposited surface area in subway and on bike

Median LDSA (µm^2^/cm^3^) on the bicycle was almost 9 times as high as in the subway (35.47 vs. 4.00, *p* = 0.042). Individual measurements are shown in Fig. 5. We did not find any significant differences in PM_10_, PM_2.5_ and PM_1_ and UFP exposure.

#### Adjusting for different velocities

Table 3 shows median mass concentrations, number concentrations, as well as LDSA adjusted for the respective velocities of the traffic mode. Adjusted PNC of ultrafine particles (pt/cm^3^) and LDSA (μm^2^/cm^3^) were significantly different in the bus when compared to the subway (2673.4 vs. 951.8, *p* < 0.001 and 1.1 vs. 0.47, *p* = 0.004, respectively). as well as in the tram compared to the subway (2031.6 vs. 951.8, *p* = 0.017/0.86 vs 0.47, *p* = 0.32, respectively).

**Table 3:** Median mass concentrations, particle number concentrations and lung deposited surface area per distance

	PM_10_ (μg/m^3^)	PM_2.5_ (μg/m^3^)	PM_1_ (μg/m^3^)	UFP (pt/cm^3^)	LDSA (μm^2^/cm^3^)
*Comparison 1*					
Subway	7.64	2.62	1.67	951.8 _*1, 2*_	0.47 _*3, 4*_
Bus	7.65	1.78	1.26	2673.4 _*1*_	1.10 _*3*_
Tram	6.38	2.29	1.40	2031.6 _*2*_	0.86 _*4*_
*Comparison 2*					
Subway	9.57 _*5*_	2.88 _*6*_	1.50 _*7*_	852.7 _*8*_	0.79 _*9*_
Bus	6.04	1.35	0.83	2304.0 _*8*_	1.40 _*9*_
Tram	8.81	2.55	1.19	1725.6	0.86
Car	2.95 _*5*_	0.69 _*6*_	0.46 _*7*_	1361.3	0.51
*Comparison 3*					
Subway	6.58	2.57	1.75	1132.8 _*10*_	0.53 _*11*_
Bus	9.00	1.98	1.30	2854.3	1.37
Tram	12.62	2.57	1.38	2295.1	1.29
Bicycle	3.71	2.11	1.64	4044.4 _*10*_	7.88 _*11*_

Concentrations of table 2 corrected for velocities of vehicles; significant differences in exposure between traffic modes are indicated by matching subscripts, e.g. velocity-adjusted exposure UFP and LDSA was significantly lower in the subway than on the bicycle in the third comparison

Adjusted PM_10_, PM_2.5_ and PM_1_ mass concentrations (μg/m^3^) were significantly higher in the subway than in the bus (9.57 vs. 2.95, *p* = 0.037, 2.88 vs. 0.69, *p* = 0.037 and 1.5 vs. 0.46, *p* = 0.016, respectively). Adjusted PNC of ultrafine particles (pt/cm^3^) as well as LDSA (μm^2^/cm^3^) were significantly different in the bus when compared to the subway (2304 vs. 852.7, *p* = 0.004 and 0.79 vs. 1.4, *p* = 0.02, respectively). The PNC of UFP and LDSA were significantly different on the bicycle when compared to the subway (4044.4 vs. 1132.8, *p* = 0.009 and 7.88 vs. 0.53, *p* = 0.004, respectively).

## Discussion

We found higher levels of PM_2.5_ and PM_1_ in the subway when compared with the bus. A similar difference between bus and subway was found in Beijing in 2015 for mass concentrations of PM_2.5_ [11]. Different results were found in Hong Kong in 2001 where air-conditioned and non-air-conditioned bus passengers were exposed to higher levels of PM_2.5_ [14].

Despite their close distance to ambient traffic, bus passengers seem to be better protected from FPM than subway commuters in Vienna. This could be due to the lack of air filtration systems in older Viennese subway trains where ventilation often occurs via open windows. Large mass concentrations of FPM have been shown in other subway systems [18], especially on platforms and in train cabins [19] with openable windows [20]. Particles in subway systems contain elements associated with mechanical origin, such as brake or wheel abrasion [21] and could be created by the trains themselves. Air quality inside subway trains can be improved by implementation of air-conditioned train cabins [22] which are being used more and more in Vienna. By contrast, PNC of ultrafine particles were significantly lower in the subway when compared to the tram. Both traffic vehicles share many characteristics in Vienna, such as large, frequently opening doors and ventilation often occurring via windows. The tram line runs considerably closer to traffic which could explain the higher PNC levels of ultrafine particles. When comparing the car with the subway we found higher levels of PM_10_, PM_2.5_ and PM_1_ in the subway despite a larger distance to traffic. Similar results were found in Milan in 2016 [10], where mean PM_10_, PM_2.5_ and PM_1_ mass concentrations were lowest in the car and in Santiago de Chile in 2014 where PM_2.5_ was lowest in the car. The study conducted in Hong Kong 2001 showed different results and found higher exposure to PM_10_ in air-conditioned taxis than in the subway [14]. Low levels of FPM in cars have previously been shown in Vienna in a different study where ventilation occurred via one partly opened window [23].

Air filtration systems in cars seem to be somewhat more effective in reducing exposure to ambient FPM. It should be noted, however, that air flow was manually set to a low level. Penetration of fine particles into vehicle cabins occurs mainly via ventilation [24] and using higher fan speeds might have led to different results.

We found that median LDSA when travelling by bike was significantly larger than in the subway. This is most likely due to closer proximity of the bike route to road traffic and the absence of any isolation of the bike rider. We did not correct for different breathing patterns. Bike commuters usually take up more pollutants due to increased minute ventilation [25] but health benefits due to physical exercise may outweigh the risks of increased uptake [26, 27].

After adjusting for different velocities, no significant differences remained between FPM exposure of bus and subway passengers, due to the higher speed of the subway and the subsequent shorter time spent inside the vehicle cabin when travelling routes of the same length. Adjusted UFP number concentrations and LDSA were significantly lower in the subway when compared to the bus due to different speeds and, in the case of the tram, the vehicle characteristics already mentioned. Comparisons of subway and car with adjusted pollution data remained significant due to similar velocities of both transport modes. The LDSA and PNC of ultrafine particles were at significantly higher levels on the bike when compared to the subway.

In general, exposure to ultrafine particles, which are mostly generated by combustion, seems to be higher near traffic (tram, bicycle), whereas coarser aerosol, either transported from street level or mechanically generated by trains, enters subway cabins.

### Reducing exposure

The higher exposure to UFP closer to one of the main sources is not surprising. Reducing UFP concentrations can be done by reducing conventional traffic and moving public transport and bike lanes away from main traffic roads. Methods such as separating the bike lane from the road via a parking lane can be enough to significantly reduce exposure to ultrafine particles [28]. The higher concentrations of FPM found in the subway are interesting. It seems as if the larger distance of underground trains to urban traffic does not necessarily protect the subway commuter from harmful pollutants. Particulate matter enters subway trains via active ventilation, open windows or open doors at the stations. The rising demand for comfortable temperatures in subway trains has already led to the implementation of air-conditioning, which, setting aside the additional energy costs, could be encouraged as it seems to be an effective way of lowering pollutants inside train cabins [22]. The same can most likely be said for trams but there are no studies confirming either hypothesis for Vienna yet. Presently, about 50% of the subway trains and 33% of the trams are air-conditioned, a situation encouraging new studies on differences in FPM and UFP exposure between those and the non-air-conditioned vehicles.

In addition, careful planning and setting of ventilation systems in new or existing subway stations should be encouraged. A recent study in Barcelona found that just changing ventilation fan directions has a significant effect on FPM concentrations on subway platforms [29]. Further studies in this area could have considerable effect, as optimizing ventilation protocols could be a cheap and quick method of lowering exposure to pollution. Air quality on platforms could also be improved by installation of platform screen doors [30].

## Conclusion

For the respective areas of Vienna, we can issue a careful recommendation for taking the subway as a substitute for the tram. Despite lower exposure to pollution, we do not recommend taking the subway instead of the bicycle as the lack of exercise may offset the positive effects of lower exposure to pollution. From a public health point of view, we cannot recommend taking a car with combustion engine as a substitute for the subway, as the additional pollution created by the vehicle would have negative health effects on other commuters. Taking the bus instead of the subway can only be recommended if the target can be reached in the same time, on the same route length the subway is the preferable transport mode. We only measured FPM, UFP and LDSA data in a small area of Vienna to contribute to the growing pool of studies concerning individual commuter exposure to particulate matter. To draw more reliable conclusions for Vienna and other cities, further investigations need to include larger parts of the public transport and road systems and consider differences in vehicle ventilation.
